# Gastric Electrical Stimulation: Role and Clinical Impact on Chronic Nausea and Vomiting

**DOI:** 10.3389/fnins.2022.909149

**Published:** 2022-05-10

**Authors:** Heithem Soliman, Guillaume Gourcerol

**Affiliations:** ^1^INSERM UMR 1073, CIC-CRB 1404, Centre Hospitalier Universitaire de Rouen, Rouen, France; ^2^Département d’Hépato-Gastro-Entérologie, Hôpital Louis Mourier, Université de Paris, Colombes, France

**Keywords:** gastric electric stimulation, gastroparesis, chronic nausea and vomiting, Enterra therapy, gastric emptying time

## Abstract

Gastric electrical stimulation (GES) is currently used as an alternative treatment for medically refractory gastroparesis. GES has been initially developed to accelerate gastric motility, in order to relieve the symptoms of the patients. Subsequent studies, unfortunately, failed to demonstrate the acceleration of gastric emptying using high-frequency stimulation – low energy stimulation although the technique has shown a clinical impact with a reduction of nausea and vomiting for patients with gastroparesis. The present review details the clinical efficacy of GES in gastroparesis as well as its putative mechanisms of action.

## Introduction

Gastroparesis is a disorder defined by delayed gastric emptying of solid food in the absence of mechanical obstruction (Camilleri et al., 2018; Schol et al., 2021a,b). Symptoms typically include early satiety, postprandial fullness, nausea, and vomiting (Wuestenberghs et al., 2019; Schol et al., 2021a,b). Severe cases can lead to impaired quality of life, dehydration, and weight loss (Yu et al., 2017). Consequently, gastroparesis has been shown to be associated with increased mortality (Jung et al., 2009; Gourcerol et al., 2022). The main etiologies are diabetes, surgery, and opioids but 40% of gastroparesis remain idiopathic (Soykan et al., 1998; Schol et al., 2021a,b; Soliman et al., 2021). The reported prevalence in the United States, and the United Kingdom is 0.02% but estimates evaluate a higher prevalence of probable gastroparesis reaching 1.8% of the general population based on suggestive symptoms (Jung et al., 2009; Rey et al., 2012; Ye et al., 2021).

The first-line treatment for gastroparesis relies on dietary modifications, with small meals and avoiding high in fat and indigestible fibers (Camilleri et al., 2013). Medical treatment is based on prokinetic drugs, most commonly dopamine antagonists such as metoclopramide or domperidone (Schol et al., 2021a). However, 30–40% of patients are refractory to a well-conducted medical treatment and will require invasive treatments (Soykan et al., 1998; Soliman et al., 2022). This review aims to discuss the efficacy, modalities, and the place of gastric electrical stimulation (GES) in the treatment of refractory gastroparesis. Putative mechanisms of action will also be discussed.

## Development and Mechanisms of Action of Gastric Electrical Stimulation

The concept of electrical gastric pacing was first developed in the 1970s, by Kelly and La Force (1972) based on studies in canine models. The first studies used a frequency in the vicinity of natural gastric slow waves and a pulse width of 100–2,000 ms (Yin et al., 2012). The long pulses are necessary to stimulate smooth muscle contractions in the gut (Chen et al., 2017). This stimulation technique was reported to “pace” the natural slow waves to the stimulation frequency. This approach was then designed to promote gastric motility and therefore accelerate gastric emptying. Most of the early studies were performed using low-frequency long pulse stimulation, i.e., “gastric pacing” with promising results in animal models, reporting normalization of gastric dysrhythmia (Lin et al., 1998). However, “gastric pacing” involves the use of long pulses and leads to high energy consumption. This resulted in the impossibility to develop an implantable pulse generator, although preliminary clinical studies using external stimulators ended up with encouraging results in gastroparesis (McCallum et al., 1998). This technique, although capable to accelerate gastric emptying, is now almost abandoned.

In the late 1990s, the Memphis group tried different stimulation parameters and revealed that a frequency four to five times higher than the intrinsic rate, associated with a shorter pulse width (<0.4 ms), allowed significant vomiting relief first in a canine model and then in patients (Familoni et al., 1997a,b). The technique was brought to clinical practice rather quickly and has been successfully applied for the treatment of gastroparesis with refractory nausea and vomiting (Forster et al., 2001). The implantable high frequency-low energy GES system, also called Enterra^®^ therapy received FDA approval for humanitarian use in 2000 for the treatment of gastroparesis (nausea and vomiting). The standard configuration has been defined as pulse trains, with a train on time set at 0.1 s and off time at 5 s, a frequency of 14 Hz, a width pulse of 330 μs, and an amplitude of 5 mA (Abell et al., 2003b).

Since GES does not accelerate gastric emptying, several mechanisms have been hypothesized to explain the antiemetic effect of this gastric neuromodulation. Indeed, the short duration of the pulse (<1 ms) failed to evoke the action potential in smooth muscle cells (Yin et al., 2012), and GES, therefore, does not modify gastric motility or gastric slow waves (Ducrotte et al., 2020). By contrast, GES has been suggested to increase the discomfort threshold to gastric distension (Gourcerol et al., 2013). This is associated with a different metabolic activity in the thalamic and caudate nuclei after GES implantation in patients (McCallum et al., 2010a). Vagal mechanisms have been reported, with a decrease in the sympathovagal balance after GES (McCallum et al., 2010a) and may explain such a sensitive effect, although a splanchnic afferent pathway has also been suggested (Gourcerol et al., 2007a; Ouelaa et al., 2012). The latter has been confirmed using a rodent model in which GES was able to modulate thoracic spinal neuronal activity in response to gastric distension (Qin et al., 2007). Last, the possible involvement of gastro-intestinal peptides release is not likely to explain the antiemetic effect of GES (Meleine et al., 2017).

## Implantation Technique and Complications

**FIGURE 1:**
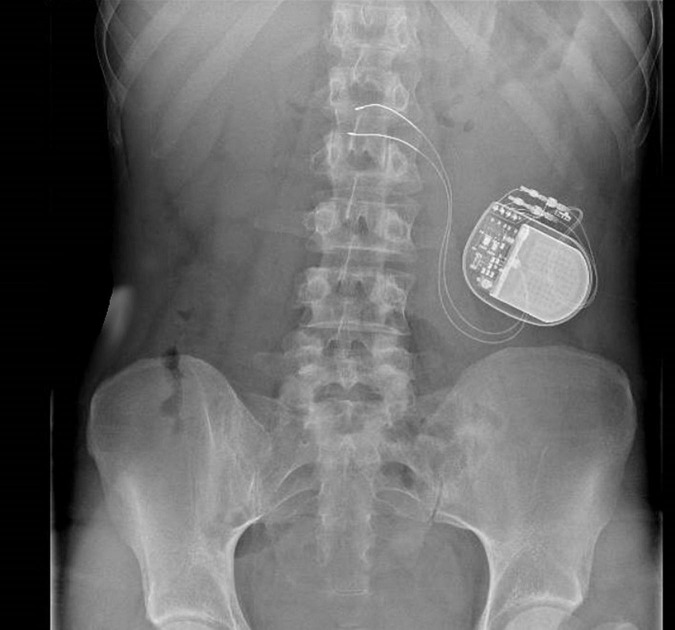
Radio of gastric electrical stimulator after implantation.

The Enterra GES is a surgical procedure with inherent risks and complications. The device is placed under general anesthesia, *via* a minimal access surgical technique (laparoscopy or laparotomy). The system consists of a pair of electrodes connected to a pulse generator. The two leads are inserted in the gastric muscularis along the great curvature of the stomach, 10 cm from the pylorus and 1 cm apart. It should be noted that the location of the leads in the stomach was determined by early studies aiming to pace gastric activity (Miedema et al., 1992). However, the location was not changed with the change of the pulse parameters. Considering the fact that the efficacy of GES is mostly sensitive, this location is not the closest to the gastric termination of the vagal nerve (Fox et al., 2000). Whether another location could improve GES efficacy or not has not been yet assessed. The distal part of the leads is then connected to the stimulator, which will be placed subcutaneously in the abdominal wall and sutured to the underlying fascia as shown in Figure 1 (Zoll et al., 2019). The programmer is then used at the end of the surgical procedure to check the impedance of the electrodes, and then adapt the parameters of the device. Patients are often hospitalized with a recovery time of 1–3 days. The battery lasts 5–10 years, and if it needs to be replaced, the electrodes do not have to be replaced (Hasler, 2009).

Bielefeldt (2017) analyzed the adverse events recorded in the manufacturer registry from 2001 to 2015. Perioperative complications are quite rare, with mainly hematoma after surgery. The complications related to the device mostly occur during the first 2 years after surgery. The most commonly reported adverse event is abdominal pain after implantation. Pain can either be reported as pain at the pocket or as an electrical shock sensation, with rarely muscle contractions. This sensation could be due to the leads, with also a role of visceral hypersensitivity. In the study of Ducrotte et al. (2020), pain was reported in 16% of patients and was always medically managed. Serious adverse events are rare. Site infection must be suspected in case of fever after surgery (6–10%), and it rarely leads to device explantation (1.5%; Abell et al., 2003b; Ducrotte et al., 2020). Intestinal occlusion has been reported and might be due to the position of the lead and the device. Thus, it is important to minimize the intraabdominal length of the leads during surgery, positioning the device in the left upper quadrant if possible (Zoll et al., 2019). Rare perforation of the leads has been reported and also requires explantation, but is very uncommon. GES safety during pregnancy has never been assessed. One case report in a female with type 1 diabetes reported a favorable outcome (Fuglsang and Ovesen, 2015).

## Clinical Efficacy of Enterra Therapy

Numerous open-label studies reported clinical improvement with GES, both in patients with diabetic and idiopathic gastroparesis (Abell et al., 2002; Lin et al., 2004; Cutts et al., 2005; Anand et al., 2007; Gourcerol et al., 2007b,^2009^; McKenna et al., 2008; Heckert et al., 2016). Most of the cohort studies evidenced that the clinical efficacy of GES was greater on nausea and vomiting as compared to other symptoms of gastroparesis, including bloating, stomach fullness, or epigastric pain. A meta-analysis including up to 600 patients showed an improvement in total symptom severity score, nausea, vomiting, early satiety, and loss of appetite (Chu et al., 2012). Long-term studies evidenced that clinical efficacy was seen even after 5 and 10 years of follow-up in more than 50% of patients in an intention to treat follow-up (McCallum et al., 2011; Gourcerol et al., 2012; Hedjoudje et al., 2020). GES was shown to reduce hospitalization requirements, nutritional status, and HbA1c (Abell et al., 2003a). The impact of GES was also reported in open-label trials in patients with chronic nausea without delayed gastric emptying (Reddymasu et al., 2010; Gourcerol et al., 2012), suggesting again that GES efficacy is not driven by the acceleration of gastric emptying.

A first 2-months randomized placebo-controlled, double-blind trial, with a crossover performed after 1 month, was published in 2003 by Abell et al. (2003b). After implantation, the stimulator was either turned ON (ON period) in the treatment arm or switched OFF (OFF period) in the control arm in a double-blinded manner. Thirty-three patients with gastroparesis were included and then randomized, half with idiopathic gastroparesis and half with diabetic gastroparesis. A significant reduction in the weekly vomiting frequency during the blinded phase was observed in the ON period compared to the OFF period. At the end of the blinded phase, patients preferred the ON period to the OFF period. Two concurrent randomized trials aimed to confirm these results in 55 patients with diabetic gastroparesis and 32 with idiopathic gastroparesis (McCallum et al., 2010b,^2013^). The design was different from the previous study since the activation of the stimulator for 1.5 months preceded a 6 months double-blinded randomized crossover period (ON/OFF for 3 months). In both trials, weekly vomiting frequency, as well as symptomatic score did not show a significant decrease among both ON and OFF periods during the crossover period. These negative results could be explained by a carryover effect due to the first 1.5 months of active stimulation since an 80% decrease in vomiting frequency was observed in this open-label prerandomization period.

A fourth large randomized controlled trial was published by Ducrotte et al. (2020). It enrolled 218 patients with diabetic, non-diabetic gastroparesis, but also patients with chronic nausea and vomiting without delayed gastric emptying. The study design was different, with the GES device remaining turned OFF for the first month after implantation and prior to randomization. Then, the randomized double-blinded period lasted 8 months, with the stimulator being switched ON or OFF after 4 months in a crossover fashion. Vomiting frequency was reduced in 30.6% of patients during the ON period compared to only 16.5% of patients during the OFF period in the crossover phase. Efficacy of GES was observed both in diabetic and non-diabetic patients, but also in patients with and without delay in gastric emptying at baseline. Likewise, gastric emptying was not normalized with GES during the ON period. Quality of life improvement was borderline significant (*p* = 0.06). Although appetite was also improved by GES, other symptoms, such as abdominal pain or bloating were not improved by GES.

Consequently, animal studies have shown an impact of GES therapy on visceral nociception, but human studies did not reveal improvement in abdominal pain (Ouelaa et al., 2012). Clinical data suggest that GES could increase the gastric maximal tolerable volume (Gourcerol et al., 2013). GES did not impact gastric compliance, but decreased the discomfort threshold to distension, suggesting an impact on the gastric visceral sensation to gastric distension. This increase in the gastric maximal tolerable volume was related to the improvement in total symptom score, and nausea and vomiting symptoms (McCallum et al., 2010a; Gourcerol et al., 2013). However, this visceral change of sensibility to gastric distension does not seem to relieve visceral pain. This sensory mechanism seems to be driven by vagal pathways, even if improvement in nausea and vomiting symptoms has also been observed in patients with previous vagotomy (McCallum et al., 2005).

Based on these studies, the level of evidence of GES in gastroparesis management was considered moderate in American gastroparesis guidelines (Camilleri et al., 2013). Likewise, in the recent UEG/ESNM expert consensus in gastroparesis, GES was rated as the third-line treatment in case of failure of metoclopramide and domperidone. However, in this report, the statement of GES as being an effective treatment of gastroparesis was rated A or A+ by only 38% and was not further endorsed (Schol et al., 2021a).

Predictive factors for GES efficacy remain unclear. No study demonstrated an impact of age, sex, or BMI, on the efficacy of the treatment. Several studies reported a better therapeutic response for patients with diabetic gastroparesis as compared to patients with idiopathic gastroparesis (Maranki et al., 2008; Richmond et al., 2015; Kim et al., 2021), although some studies did not observe different outcomes (Anaparthy et al., 2009; Gourcerol et al., 2009). Postoperative gastroparesis is also associated with a better response to GES therapy, except for patients with partial gastrectomy (McCallum et al., 2005; Gourcerol et al., 2009). Previous use of opioid treatment, and patients having pain rather than nausea vomiting have worst outcomes with GES (Maranki et al., 2008). Finally, delayed gastric emptying is not predictive of a better response to GES. Therefore, GES has been shown to be effective in patients with gastroparesis as well as patients with chronic nausea and vomiting without delayed gastric emptying (Gourcerol et al., 2009; Hejazi et al., 2011; Hou et al., 2012; Ducrotte et al., 2020).

## Cost-Effectiveness of Gastric Electrical Stimulation

A major limit to the development of the Enterra therapy^®^ has been the cost of the device since the stimulator itself costs approximately $15,000 per patient. Data on health costs related to gastroparesis are scarce. One study in the United States showed that gastroparesis-induced costs reached up to $34,585 per patient per year in 2013, mainly driven by hospitalizations (Wadhwa et al., 2017). This study revealed a 313% increase from 1997 to 2013 in the number of hospital discharges with gastroparesis as a principal diagnosis. This led to a national bill increase for gastroparesis of 1,026%, reaching $568 million in 2013. Based on these findings, three studies assessed the cost-effectiveness of GES.

The first one performed in the United States investigated the economic impact of GES in nine patients (Cutts et al., 2005). After 3 years, this study showed that GES decreased hospitalizations and direct healthcare costs as compared to conventional medical treatment. The yearly costs dropped from $83,000 at baseline to $22,000 at 3 years whereas it only decreased to $63,000 in the group treated with conventional treatment. Larger studies in Europe showed similar results. A second study performed in Denmark evaluated the cost-utility of the device on 30 diabetic patients (Klinge et al., 2017). The healthcare costs reached €16,611 per year before GES implantation. These costs fell to €10,000 in the first year, and to €104 in the second year. The last multicentric study was performed on 172 patients in France and is an extension of the study of Ducrotte et al. (2020) and Gourcerol et al. (2020). In this study, GES reduced healthcare costs from €7,915 to €4,928 per patient and per year. GES reduced direct costs, mostly driven by hospitalization rate, but also indirect healthcare costs, including time off work (Gourcerol et al., 2020). Greater savings were observed for diabetic patients compared to patients with idiopathic gastroparesis. Altogether, these studies demonstrated that GES is cost-effective since savings cover the price of the device a few years after its implantation.

## Future Direction

An approach to select better responders to GES could be the use of temporary electrical stimulation. This technique involves either the electrode placement through a G tube (Elfvin et al., 2007, 2011; Andersson et al., 2011) or using the endoscopic placement of cardiac pacing leads into gastric mucosa (Ayinala et al., 2005; Daram et al., 2011). The latter technique involves an inner bipolar electrode pacing lead and is left like a nasogastric tube. Electrodes are then connected to an external standard device, in a shirt pocket and adjusted with the same parameters as the Enterra therapy. In a randomized trial, temporary stimulation led to a reduction in the vomiting frequency, even if not significant due to some limitations of the study design (Abell et al., 2011). This technique has also been evaluated over 551 patients suffering from symptoms of gastroparesis and was effective in patients with or without delayed gastric emptying (Singh et al., 2015). Temporary GES revealed improvement in vomiting, nausea, and total symptom severity score. Finally, the study by Corvinus et al. (2018) revealed that patients who had a clinical improvement with temporary GES were responders to GES therapy. Moreover, the location of the implantation of electrodes can be easily changed with temporary stimulation. Thus, this technique could help define the best location for the electrodes according to gastric innervation (Fox et al., 2000). The main limitation of temporary GES is that the technique is not currently commercially available.

Other improvements could be a better adjustment of GES settings. Indeed, a subset of patients will need to change the settings of the device to reach the efficacy of GES therapy, with an increase in the current or the pulse frequency (Abidi et al., 2006). On the other hand, patients might have a sustained improvement a few weeks after stopping stimulation. This sustained efficacy is suggested by the carryover effect observed in the randomized trials with permanent or temporary GES (McCallum et al., 2010b,^2013^; Abell et al., 2011). Thus, intermittent GES, alternating a few weeks on, and a few weeks off stimulator might be an interesting strategy. Moreover, taking advantage of this carryover effect could salvage battery life.

Recently, pyloric targeting therapies have emerged in the treatment of refractory gastroparesis, especially gastric peroral endoscopic myotomy (G-POEM; Jacques et al., 2019b). Indeed, GES does not accelerate gastric emptying while pyloric targeted therapies do (Wuestenberghs and Gourcerol, 2021). Open labeled studies have shown clinical improvement and acceleration of gastric emptying in 56–70% of patients after G-POEM (Jacques et al., 2019a; Ragi et al., 2020; Vosoughi et al., 2021). Whether GPOEM is more effective than GES to relieve nausea and vomiting associated with gastroparesis remains unknown (Soliman et al., 2022). One study aimed to compare GES and G-POEM and concluded that G-POEM could have a better long-term efficacy (Shen et al., 2020). However, this study was not performed specifically on gastroparesis associated with nausea and vomiting, while these symptoms are targeted by GES therapy. Furthermore, some authors suggested that GES and G-POEM could be complementary, treating different mechanisms of gastroparesis (Parkman, 2020). Studies have first assessed the impact of the addition of pyloric surgery to GES implantation (Sarosiek et al., 2013; Davis et al., 2017). This combination therapy led to an acceleration in gastric emptying, as compared to GES alone, but also in better symptomatic improvement (Zoll et al., 2020). Finally, the combination of GES and G-POEM has been studied in 22 patients and also appeared as safe and effective (Strong et al., 2019). Further studies are still needed to define the best therapeutic strategy and to select the best treatment for each patient.

## Conclusion

High-frequency GES with Enterra therapy is a safe and effective technique to relieve nausea and vomiting in patients refractory to medical treatment. The clinical efficacy reaches 50–70% of patients with long-term efficacy of up to 10 years. Mechanisms of action remain poorly understood although the symptomatic improvement is not related to gastric emptying acceleration. Future directions encompass the spread of temporary GES in routine care and comparison and/or combination with other existing concurrent techniques, including pyloromyotomy.

## Author Contributions

HS and GG contributed to the design of the manuscript. HS wrote the first draft of the manuscript. GG critically reviewed the manuscript. Both authors read and approved the final version.

## Conflict of Interest

The authors declare that the research was conducted in the absence of any commercial or financial relationships that could be construed as a potential conflict of interest.

## Publisher’s Note

All claims expressed in this article are solely those of the authors and do not necessarily represent those of their affiliated organizations, or those of the publisher, the editors and the reviewers. Any product that may be evaluated in this article, or claim that may be made by its manufacturer, is not guaranteed or endorsed by the publisher.
